# Annotating and quantifying pri-miRNA transcripts using RNA-Seq data of wild type and *serrate-1* globular stage embryos of *Arabidopsis thaliana*

**DOI:** 10.1016/j.dib.2017.10.019

**Published:** 2017-10-12

**Authors:** Daniel Lepe-Soltero, Alma Armenta-Medina, Daoquan Xiang, Raju Datla, C. Stewart Gillmor, Cei Abreu-Goodger

**Affiliations:** a Laboratorio Nacional de Genómica para la Biodiversidad (Langebio), Unidad de Genómica Avanzada, Centro de Investigación y de Estudios Avanzados (CINVESTAV), Irapuato, Guanajuato, Mexico; b Plant Biotechnology Institute, National Research Council, Saskatoon, Saskatchewan, Canada

## Abstract

The genome annotation for the model plant *Arabidopsis thaliana* does not include the primary transcripts from which *MIRNAs* are processed. Here we present and analyze the raw mRNA sequencing data from wild type and *serrate-1* globular stage embryos of *A. thaliana*, ecotype Columbia. Because *SERRATE* is required for pri-miRNA processing, these precursors accumulate in *serrate-1* mutants, facilitating their detection using standard RNA-Seq protocols. We first use the mapping of the RNA-Seq reads to the reference genome to annotate the potential primary transcripts of *MIRNAs* expressed in the embryo. We then quantify these pri-miRNAs in wild type and *serrate-1* mutants. Finally, we use differential expression analysis to determine which are up-regulated in *serrate-1* compared to wild type, to select the best candidates for *bona fide* pri-miRNAs expressed in the globular stage embryos. In addition, we analyze a previously published RNA-Seq dataset of wild type and *dicer-like 1* mutant embryos at the globular stage [1]. Our data are interpreted and discussed in a separate article [2].

**Specifications Table**

**Table:** 

Subject area	*Biology*
More specific subject area	*Plant biology*
Type of data	Tables
How data was acquired	RNA-Seq from an Illumina HiSeq 2000 and previously published *dcl1-5* raw data from the Gene Expression Omnibus (GEO) accession GSE25404[1]
Data format	*Raw and analyzed data*
Experimental factors	Total RNA was extracted from pools of ~80 globular stage (32–64 cell) embryos isolated at 72 hours after pollination
Experimental features	*Arabidopsis thaliana* wt and *se-1* embryos at the globular stage, with two biological replicates
Data source location	*Not applicable*
Data accessibility	*Data is available as*Supplementary file 1*and at NCBI GEO accession*GSE100450

**Value of the data**

• This is the first study to directly identify *MIRNA* genes expressed in early embryos of plants.
• We provide an annotation file with 318 *MIRNA* gene models*,* including 77 predicted from the RNA-Seq data, that is useful for others interested in *MIRNA* gene regulation in *Arabidopsis*.
• Our high-quality globular stage transcriptomes of wild type and *serrate-1* embryos will be valuable for other studies of gene regulation in early embryogenesis.

## 1 Data

We generated RNA-Seq data for globular stage *Arabidopsis thaliana* embryos from two genotypes: *serrate-1* (*se-1*) mutants, and wild type (wt), both in the Columbia ecotype. We then inferred *MIRNA* primary transcripts expressed at the globular stage by aligning RNA-Seq reads to the *Arabidopsis* genome, assembling and manually curating gene models, and analyzing differential expression. As an independent profile of *MIRNA* transcripts in embryos, we analyzed a previously published RNA sequencing experiment using wt Columbia and *dicer-like 1* (*dcl1-5*) embryos [1]. We provide the raw data, predicted pri-miRNA gene models, quantification of all genes in both experiments, and differential expression results.

## 2 Experimental design, materials and methods

Two biological replicates of wt and *se-1* of about 80 embryos each at the 32–64 cell (early to mid-globular) stage were obtained, the RNA isolated, amplified and sequenced as described previously [2]. Illumina HiSeq 2000 sequencing yielded 101 nt paired-end reads with over 20 million reads per library (Table 1). Raw data files are available through the NCBI Gene Expression Omnibus (GEO, accession GSE100450). Raw data from the *dcl1-5* RNA-Seq experiment was downloaded from GEO (accession GSE25404), consisting of a single replicate of *dcl1-5* mutant and Col-0 (wt) early globular embryos with 36 nt reads obtained on an Illumina Genome Analyzer II [1].

**Table 1:** RNA sequences obtained by Illumina sequencing.

**Sample**	**Raw reads**	**Quantified reads**
WT1	20,751,215	18,894,161 (91.05%)
WT2	21,623,459	19,362,653 (89.54%)
se1	20,708,775	18,580,516 (89.72%)
se2	22,984,350	20,882,004 (90.85%)

The paired-end reads from the wt and *se-1* libraries were mapped using HISAT2 [3] with default settings except for an intronic length suited for Arabidopsis (--max-intronlen 900). The default intronic length (--max-intronlen 500000), tuned for mammalian genomes, resulted in many reads falsely mapping across several genes. The 36 nt reads from the wt and *dcl1-5* libraries were cleaned using cutadapt [4] v1.13 to remove adaptors, polyA and polyT sequences from the 3′ and 5′ ends (respectively), and low-quality bases and flanking Ns were trimmed from individual reads. Finally, any read shorter than 18 nt or with more than three internal Ns was discarded. The full parameters for cutadapt were: -q 6 -a ATCTCGTATGCCNNNNNNNNNNNNNNNNNNNNNNNN -a "A{36}" -g NNNNNNNNNNNNNNNNNNNNNNNNAGTCCGACGATC -g "T{36}" --trim-n --max-n 3 -m 18.

The resulting cleaned reads were mapped using the Bowtie [5] short read aligner allowing up to 2 mismatches within a 25 nt seed sequence, and only uniquely mapped reads were retained (-l 25 -n 2 -m 1) (Table 2). Both transcriptomes were mapped to the reference TAIR10 assembly of *Arabidopsis thaliana*, downloaded from The Arabidopsis Information Resource (ftp://ftp.arabidopsis.org/home/tair/Sequences/whole_chromosomes/).

**Table 2:** RNA sequences from GSE25404.

**Sample**	**Raw reads**	**Quantified reads**
WT (Col-0) (SRR074122)	21,413,867	5,153,766 (24.06%)
dlc1-5 (SRR074123)	22,578,840	4,047,267 (17.92%)

The Araport11 reference annotation from the Arabidopsis Information Portal [6] contains the coordinates of pre-miRNA hairpins, but does not contain information regarding the pri-miRNA transcripts. In order to predict genome wide pri-miRNA transcript coordinates, Cufflinks [7] was used to assemble and merge putative pri-miRNA transcripts from the *se-1* and wt Col RNA-Seq read alignments. Cufflinks was first run for each library (--overlap-radius 1 --library-type fr-unstranded). The predictions were then merged (cuffmerge -s) using the TAIR10 genome assembly as reference. Out of the 325 miRNAs in the Araport11 annotation, 77 overlapped with a predicted Cufflinks gene model. All these predictions were manually verified, with only 4 of them requiring individual adjustments to better reflect the disposition of the reads from the RNA-Seq libraries, and to resolve overlapping conflicts. The main limitation of this approach is that pri-miRNAs can only be assembled if they are expressed in the sampled conditions (in this case, for early globular embryos).

Overlaps with, or even proximity to protein-coding genes can make it difficult to establish the appropriate gene model of a pri-miRNA. Due to this, the pri-miRNA predictions were divided into four groups: intergenic (G1), between 1–400 bp away from a protein-coding gene (G2), overlapping with a protein-coding gene (G3, divided into G3A if the overlap includes the pre-miRNA or G3B otherwise) and overlapping with a non-coding gene (G4); see Table 3. The 77 Cufflinks predictions were distributed amongst the pri-miRNA groups as follows: 28 in G1, 7 in G2, 0 in G3A, 37 in G3B and 5 in G4. There were 56 pre-miRNAs that overlapped with 54 protein-coding genes (G3A group). These pre-miRNAs were assigned to a pri-miRNA gene model identical to the overlapping protein-coding gene. In all other cases where no Cufflinks prediction was available, the pri-miRNA was kept the same as the pre-miRNA annotation from Araport11.

**Table 3:** Groups of miRNAs according to their position relative to other annotation in the Arabidopsis genome.

**Group**	**Description**	**Number of pre-miRNAs in Araport11**	**Predicted pri-miRNA gene models**	**Gene models from Araport11**	**Gene models with two pre-miRNAs**	**Pri-miRNAs gene models in final annotation**
G1	Intergenic region	222	28	166	2	194
G2	Closest protein-coding gene is 1–400 bp away	39	7	19	0	26
G3A	Overlap of pre-miRNA with protein-coding gene	56	0	54^a^	3	54
G3B	Overlap of pri-miRNA with protein-coding gene	0	37	0	1	37
G4	Overlaps with non-coding gene	8	5	2	1	7
**Total**		**325**	**77**	**241**	**7**	**318**

a The overlapping protein gene model was used instead of the pre-miRNA coordinates.

A final annotation file with the newly predicted pri-miRNA gene models, in addition to all the gene models from Araport11, considering a total of 318 pri-miRNA genes and 27,562 protein-coding genes, was employed for the quantification of all the RNA-Seq libraries and is available as Supplementary file 1. Quantification of reads using this annotation file was performed in R with the function *featureCounts* from the *Rsubread* package [8]. Multi-mapping reads were counted (countMultiMappingReads=TRUE) and only primary alignments were allowed (primaryOnly=TRUE). Additionally, reads were assigned to the feature with the largest number of overlapping bases (largestOverlap=TRUE) and a minimum mapping quality score of 10 was required (minMQS=10) for a read to be counted.

Finally, the *edgeR* [9] package was used to perform the differential expression analysis of both the *se* and *dcl1* experiments, using the raw counts with no prior filtering. A tagwise dispersion was calculated for *se*, but since no replicates are available for the *dcl1* experiment, the Biological Coefficient of Variation was fixed to 0.4, as recommended by the edgeR manual. To test for differential expression, quasi-likelihood F-tests and likelihood ratio tests were performed for the *se* and *dcl1* experiments, respectively. In total, 6951 genes were upregulated in the *se-1* mutant and 7138 were downregulated with an FDR < 0.05, and 125 genes were upregulated in *dcl1-5* and 138 downregulated with an FDR < 0.05 (Table S3 from reference [2]).

Of the 318 annotated pri-miRNAs, 100 were deemed differentially expressed (FDR < 0.05) in *se-1*. Of those, 73 were upregulated (G1: 22, G2: 6, G3A: 17, G3B: 25, G4: 3) and 27 were downregulated (G1: 5, G2: 1, G3A: 14, G3B: 7, G4: 0). Because *SERRATE* participates during pri-miRNA processing, they should be up-regulated in *se-1.* To evaluate which of the pri-miRNA groups behaved as expected in the *se-1* mutant, a one-sided Wilcoxon rank sum test was done with the *wilcox.test* function from the *stats* package in R. The resulting p-values were G1: 0.0016, G2: 0.017, G3A: 0.14, G3B: 0.000017, and G4: 0.04, suggesting that in most cases the gene-models do reflect the properties of a pri-miRNA (except for the G3A category, where the gene models are taken from protein-coding genes). The average values of expression and log_2_FC for all genes, including pri-miRNAs and the median log_2_FC for each of the miRNA groups, is plotted in Fig. S2 from Ref. [2].

In *se-1*, 133 miRNAs have at least 4 accumulated reads from the libraries. Of these, 100 are differentially expressed (FDR < 0.05). For *dcl1-5*, 121 miRNAs have at least 4 accumulated reads from the libraries. A summary of the miRNAs that were detected in both the *se-1* and *dcl1-5* experiments is detailed in Table 4 and a summary of their differential expression behavior is shown in Table 5, Table 6.

**Table 4:** Common pri-miRNAs detected with at least 4 reads in the *se-1* and *dcl1-5* experiments.

	***dcl1-5 (121 miRNAs)***
***se-1 (133 miRNAs)***	117
***se-1*****(FDR < 0.05) (100 miRNAs)**	90

**Table 5:** Behavior of differentially expressed pri-miRNAs in *se-1* and *dcl1-5*.

117 common pri-miRNAs (no FDR filter)		***dcl1-5***	
		**Upregulated**	**Downregulated**
***se-1***	**Upregulated**	47	39
	**Downregulated**	11	20

**Table 6:** Behavior of differentially expressed pri-miRNAs in *se-1* (FDR < 0.05) and *dcl1-5 (no FDR filter)*.

90 common pri-miRNAs (FDR < 0.05 for *se-1*)		***dcl1-5***	
		**Upregulated**	**Downregulated**
***se-1***	**Upregulated**	37	31
	**Downregulated**	7	15
